# Compensatory Plasticity in the Deaf Brain: Effects on Perception of Music

**DOI:** 10.3390/brainsci4040560

**Published:** 2014-10-28

**Authors:** Arla Good, Maureen J. Reed, Frank A. Russo

**Affiliations:** Ryerson University, 350 Victoria St. Toronto, ON M5B 2K3, Canada; E-Mails: mreed@psych.ryerson.ca (M.J.R.); russo@psych.ryerson.ca (F.A.R.)

**Keywords:** compensatory plasticity, deaf, music

## Abstract

When one sense is unavailable, sensory responsibilities shift and processing of the remaining modalities becomes enhanced to compensate for missing information. This shift, referred to as compensatory plasticity, results in a unique sensory experience for individuals who are deaf, including the manner in which music is perceived. This paper evaluates the neural, behavioural and cognitive evidence for compensatory plasticity following auditory deprivation and considers how this manifests in a unique experience of music that emphasizes visual and vibrotactile modalities.

## 1. Introduction

A number of researchers have explored how sensory deprivation in one modality may affect the development of the remaining modalities. When one sense is unavailable, sensory responsibilities shift, and processing of the remaining modalities becomes enhanced. This shifting of responsibilities appears to be compensatory in nature and has thus come to be referred to as compensatory plasticity. For example, as the auditory sense is typically responsible for gathering information and monitoring events in the surrounding environment, deaf individuals tend to compensate with enhanced visual processing for events in the peripheral field of vision [1,2,3,4]. Enhancements such as these do not appear to be due to decreases in absolute sensory thresholds as much as changes in the manner in which the sensory information is processed [5]. This results in a unique sensory experience that influences the manner in which music is perceived.

Frequently cited definitions of music, such as “organized sound” [6] or “an ordered arrangement of sounds and silences…” [7] emphasize the cultural supremacy of sound in the domain of music. These definitions imply that the deaf population has limited access to music, as well as to the emotional aspects of speech, which also tend to be musical (e.g., speech prosody, vocal timbre). However, definitions of music and emotional speech that focus exclusively on sound fail to incorporate multi-modal aspects, such as body movements, facial expressions and vibrotactation. These multi-modal aspects make music and emotional speech accessible to people of all hearing abilities.

Changes in brain, cognition and behavior following auditory deprivation are typically interpreted from one of two complementary perspectives: neuro-developmental or cognitive. The neuro-developmental perspective focuses on changes that are due to a structural reorganization of the pathways in the brain. The cognitive perspective focuses instead on changes that are due to differences in the recruitment of attention and available resources. The following two sections will review research from neural developmental and cognitive perspectives, in turn. These sections are followed by a consideration of non-auditory elements in music that may be perceived differently following auditory deprivation.

## 2. Compensatory Plasticity: The Neuro-Developmental Perspective

**Figure 1:**
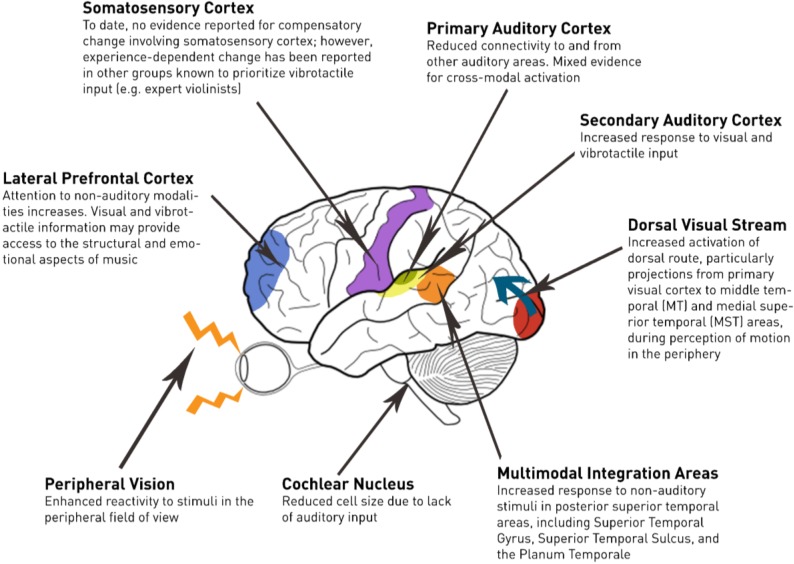
Changes in the deaf brain relevant to music perception.

Figure 1 provides a neuro-anatomical view of subcortical and cortical changes occurring following deafness. A subset of these changes has been described as a degeneration of the auditory pathways over time [8,9]. For example, Saada *et al*. [9] report that the cochlear nucleus of the deaf white cat (a congenitally deaf strain) is half the size of typically hearing cats. The deaf white cat is considered an animal model for prelingual deafness given that it shows an absence of brainstem-evoked potentials to clicks presented up to 120 dB SPL [10]. Moore *et al.* [8] noted a similar pattern in human participants, wherein the size of cells in the cochlear nucleus of individuals with profound hearing loss was reduced by up to fifty percent relative to hearing controls. Moreover, there was a robust negative correlation observed between cell size and the duration of profound deafness.

In contrast to the gross morphological changes observed at the level of the brain stem, the total volume of grey and white matter within the primary auditory cortex does not appear to be affected by deafness ([11]; but see [12]). However, congenitally deaf individuals show an increase in grey-white ratios relative to their hearing counterparts. This difference in ratios is likely the result of reduced myelination and/or fewer fibers that project to and from the auditory cortex. This finding is consistent with the view that sensory deprivation leads to changes in cortical connectivity due to axonal pruning [13]. These changes may be interpreted as progressive, rather than degenerative; *i.e.*, compensations for sensory deprivation.

### 2.1. Auditory System Response Changes Following Deprivation

The majority of evidence for crossmodal activation of the auditory cortices in the deaf brain involves visual activation of the secondary auditory cortex. When profoundly deaf individuals are asked to view sign language, activity is observed in the secondary auditory cortex [14]. Hand motions without a lexical meaning activate areas of the secondary auditory cortex in deaf participants [15]. Strikingly, even moving dot patterns activate the secondary auditory cortex in deaf participants [15,16,17]. Sung and Ogawa [18] suggest that some of the reorganization in secondary auditory areas may also be owed to practice with visual language.

Tactile stimulation also elicits activation in the secondary auditory cortex of individuals who are deaf. When vibration is presented to the palms and fingers, activation of the secondary auditory cortex is greater and more widespread in deaf participants than in hearing participants [19,20].

Debate exists regarding whether the primary auditory cortex (area roughly corresponding to Heschl’s gyrus) adapts itself to processing information from the non-auditory modalities. Animal research involving the deaf cat has found limited evidence for reorganization in the connectivity of the primary auditory cortex [21]. In human work, some researchers have found evidence for non-auditory activation in the primary auditory cortex during visual processing [17,22], while others have shown activation during somatosensory processing [19,23]. In contrast, Hickok *et al*. [24] found no primary auditory cortex activation using magnetoencephalography (MEG) and functional magnetic resonance imaging (fMRI) in a single deaf participant during visual and somatosensory stimulation.

The reorganization of the auditory brain following deafness has been causally linked to enhanced visual discrimination in an animal model. Congenitally deaf cats are known to have superior visual localization in the peripheral field and to have lower visual movement detection thresholds. Lomber, Meredith, and Kral [25] showed that deactivation of the posterior auditory cortex selectively eliminates the enhanced visual localization, whereas deactivation of the dorsal auditory cortex eliminates the enhanced visual motion detection.

### 2.2. Non-Auditory System Changes Following Auditory Deprivation

Behavioral studies have not been able to show that auditory deprivation leads to enhancements in the absolute sensitivity of the spared modalities. Deaf and hearing populations demonstrate comparable thresholds for visual brightness and contrast [26,27]. Imaging studies have also been unable to reveal definitive evidence for differences in activation of the primary visual cortex between deaf and hearing participants (e.g., [16]). Nonetheless, deaf individuals do show differences in visual processing of motion [28] particularly in the periphery [29]. Research involving the deaf cat found evidence to suggest that extrastriate visual areas, such as those involved in visual motion processing, undergo cross-modal reorganization [30]. In human participants, motion selective areas of the dorsal visual stream, such as the medial temporal (MT) and medial superior temporal (MST), are more activated in deaf individuals compared to hearing individuals when visual motion is presented to the peripheral field of view [31,32].

Given their reliance on tactile input, we might expect deaf individuals to have increased activation of the somatosensory cortex relative to hearing individuals. The somatosensory representation of the digits in the left hand of expert string players has famously been found to be larger than that of controls [33]. Presumably, this increased representation is owed to the importance of vibrotactile feedback for string players. However, despite the importance of vibrotactile feedback for deaf individuals, no corresponding evidence has been reported to date.

### 2.3. Multimodal Integration Areas

Many cortical changes occur beyond the primary sensory cortices [34]. There is agreement in the compensatory literature that the multimodal integration areas of the brain tend towards prioritization of input from the in-tact modalities [13]. Posterior areas of the superior temporal lobe are well-defined multi-sensory integration areas [35] that have also been associated with language processing [36]. In deaf individuals, these multimodal integration areas, including the superior temporal gyrus [18,31,37], superior temporal sulcus [38] and the planum temporale [15,38], are activated more strongly compared to hearing individuals during visual stimulation.

### 2.4. Limitations of Neuro-Developmental Perspective

The neural perspective leaves several questions unanswered regarding compensatory plasticity. Foremost among these is the evidence for compensatory changes that are not limited to the congenitally deaf population. A critical period for cross-modal plasticity following sensory deprivation exists [39]. For example, a functional adjustment in the region of the auditory cortex associated with language processing appears to depend on a critical period that ends as early as two years of age [38]. However, there have been examples of behavioural changes in sensory processing due to deafness in individuals with much later onsets of deafness [40]. This capacity for change beyond the critical period suggests that compensation may also rely to some extent on cognitive strategies, including the recruitment of increased attentional resources and a redirection of attention to non-auditory modalities. The locus of control for such effects on attention, at least during the early stages of deafness, is likely to be the lateral prefrontal cortex [41].

## 3. Behavioral Compensation: The Cognitive Perspective

Behavioral outcomes are not simply a manifestation of neural plasticity alone. The compensation may also involve top down processes, including increased attention directed towards the remaining modalities. Studies that fail to find neural differences in response to non-auditory stimuli among deaf and hearing individuals often utilize passive processing of visual stimuli [24]. More clear-cut support for cross-modal activation has been seen under attentionally demanding conditions [42].

Complex behavioural tasks that require attention redirection consistently reveal differences between deaf and hearing individuals (for a review, see [5]). For example, deaf participants are faster and more efficient in completing visual search tasks than hearing participants [43]. Deaf participants also appear to engage in more efficient forms of processing visual information. Stivalet *et al.* [43] asked hearing and deaf participants to detect letter targets among distractors. Differential search patterns were reported for each group. Hearing participants showed search patterns that were asymmetric across conditions compared to deaf participants, whose search patterns were symmetric and more efficient overall.

Interference effects from visual distraction provide additional evidence of altered attention redirection in deaf individuals [44]. Deaf individuals show a shift in the spatial distribution of attention from the center to the periphery [44,45,46]. Parasnis and Samar [46] conducted a stimulus detection task in which cues were presented to provide information regarding target location. Some of the trials contained a complex task-irrelevant distractor that participants were asked to ignore. Results showed that deaf participants were more adept at redirecting their attention between spatial locations in the periphery than were hearing participants.

### 3.1. Enhanced Visual Attention to the Periphery

Deaf individuals appear to allocate increased visual attention to the periphery relative to hearing individuals, while no difference appears to exist between groups in visual attention to central targets [1,2,3,4,44]. Imaging studies by Bavelier and colleagues [31,32] have revealed enhanced recruitment of motion-selective areas (middle temporal/medial superior temporal) for deaf individuals in tasks requiring the detection of luminance changes in the periphery. Behavioural outcomes are also consistent with imaging results. When asked to attend to visual stimuli in the periphery, the deaf participants outperform their hearing counterparts. However, deaf and hearing participants do not show a significant difference in outcomes when asked to attend to visual stimuli in the central field of view.

Neville and Lawson [2] found that deaf participants more accurately reported motion direction than did hearing participants. Bosworth and Dobkins [47,48] found a similar pattern of performance on a visual motion discrimination task. Interestingly, the latter study showed a strong right visual field advantage for deaf participants, suggesting that the left hemisphere, which classically includes language areas, is preferentially recruited in deaf individuals to support the processing of visual motion. These findings support the view that neural plasticity and attention may both contribute to some of the perceptual advantages observed in deaf participants.

As noted earlier, some of the visual processing advantages of deaf individuals may also stem from frequent practice in the use of visual language [18]. Sign language users must learn how to be especially sensitive to hand and body movements, especially when they occur in peripheral space. Neville and Lawson [3] compared the detection of apparent motion in deaf signers, hearing signers and hearing non-signers. Findings illustrated that the deaf signers were faster than the other two groups in reporting the direction of motion of the target stimuli in the peripheral field of vision. Nonetheless, individuals who have practice with sign languages, regardless of hearing ability, may recruit resources from the left hemisphere language regions when processing visual motion [3]. Taken together, these studies strongly suggest that fluency with sign language may be partly responsible for enhancements in motion perception observed in deaf individuals.

### 3.2. Enhanced Visual Attention to Facial Features

Individuals who are deaf demonstrate enhanced capability to understand speech through the visual modality. For example, deaf individuals demonstrate higher levels of proficiency in lip reading, or speech reading, compared to their normal hearing counterparts [49,50]. Deaf sign language users also demonstrate an enhanced ability to detect even subtle differences in facial expressions, compared to non-signers [51]. This is not surprising in that facial expressions serve important grammatical and semantic roles in sign language [52]. The cognitive perspective of compensation would argue that the extra practice with facial details should lead to enhancements in facial recognition ability. Bettger *et al.* [40] examined this hypothesis by asking participants to discriminate pictures of human faces under varied conditions of lighting and positioning. They found that deaf signers performed significantly better than hearing participants on this task, especially in situations of poor lighting. To determine whether outcomes were due to auditory neural enhancement or exposure to sign language, Bettger *et al.* [40] repeated the experiment with hearing participants who were native signers and deaf participants who adopted sign language later in life. Both signing groups (deaf and hearing) and the deaf late signers demonstrated trends toward greater facial discrimination than the hearing non-signers. These results suggest that practice with sign language, rather than auditory neural enhancement alone, leads to greater facial discrimination. Deaf individuals who were exposed to sign language later in life still demonstrated superior facial recognition compared to hearing non-signers, suggesting that there may be no critical period to develop this aptitude.

### 3.3. Enhanced Attention to Vibrotactile Stimuli

Deaf individuals show some processing advantages for haptic and vibrotactile stimuli [53,54]. Levänen and Hamdorf [54] explored the vibrotactile sensitivity of congenitally deaf participants by using sequences of vibration. They found an enhanced ability in the congenitally deaf to detect suprathreshold frequency changes in vibrotactile stimulation. However, on the basis of this study alone, it is not clear whether this enhancement was due to compensatory neural plasticity or changes in attention due to practice.

## 4. Compensation Summary

Though it is not always clear whether the deaf brain compensates through neuro-development changes, changes in attentional focus or some combination, all of the available evidence suggests that there is a compensatory shift in sensory responsibilities. This shift results in a non-auditory sensory experience that is unique to individuals who are deaf. This unique sensory experience may enhance the perception of non-auditory elements of music.

## 5. Non-Auditory Elements of Music and the Deaf Experience

### 5.1. Visual Elements

The appreciation of music does not rely exclusively on sound. While music is generally focused on the auditory modality, the movements of performers, including hand gestures, body movements and facial expressions, influence an audiences’ perceptual and aesthetic assessment of music [55,56,57,58].

Body movement and facial movements made by performers often provide structural information about music. For example, judgments of musical dissonance can be influenced by visual aspects of a guitar performance [56]. Facial movements made by singers provide information about the size of the pitch interval between notes [56]. Video-only presentations of melodic intervals are scaled accurately by observers and do not appear to depend on musical or vocal experience [59]. Video-based tracking of sung intervals has demonstrated that larger intervals involve more head movement, eyebrow raising and mouth opening [59].

Musical performers also use facial and body movements to intentionally convey emotional aspects of music. Thompson and Luck [57] used a motion capture system to explore the ability of a musician to convey emotion through movements of the face and body. When musicians were asked to play with an exaggerated emotional expression, they found an increase in the amount of bodily movement.

The visual aspects of music may be especially exploited by a deaf audience. For example, the enhanced ability possessed by the deaf to discriminate faces becomes especially relevant when faces are partially occluded [40] as is often the case in live or recorded music. In addition, the increased ability for visual discrimination in the periphery may have implications for how the deaf experience visual aspects of music, beyond the more obvious aspects in the central field of view.

### 5.2. Vibrotactile Elements

Sound at its most basic level is airborne vibration [60]. Given this physical aspect, loud sound may elicit vibrations that may be transmitted through walls, floors and furniture. These physical vibrations reach the hair cells of the cochlea at different frequencies. Each hair cell of the cochlea is most sensitive to a specific frequency; called the characteristic frequency [61]. The auditory cortex receives signals fired from the frequency-specific nerve fibers of the cochlea. These auditory frequencies, or pitches, activate specific areas of the auditory cortex [62]. Together, these different auditory pitches create melody, harmony and timbre. The vibrotactile receptors in the skin are biomechanically similar to the cochlear hair cells. However, receptors in the skin do not process the same range of frequencies as the cochlear hair cells; instead different classes of receptors respond to different frequency ranges [63].

A growing body of empirical research has provided a better understanding of the ability to perceive musical information through vibrotactile sources alone. For example, one of the basic elements found to convey emotion in music is tempo [64]. This term refers to the overall rate of presentation, conventionally measured in beats per minute (BPM). Tactile representation of the tempo using differing inter-pulse intervals can be easily represented through naturally occurring or amplified vibrations produced by voices and musical instruments. There are strong associations between tempo and emotion; happy songs tend to be associated with faster tempos, while sad songs tend to be associated with slower tempos [65]. Recent research in our own lab involving vibrotactile reproductions of music has found that deaf individuals show similar associations with tempo [66].

Though the tempo can be easily accessed through vibration, pitch perception proves to be more limited. Discrimination thresholds for frequencies are much larger for vibrotactile than for auditory presentations, and discriminations are limited to frequencies below about 1000 Hz [67,68]. However, the majority of these findings are based on research involving hearing participants. As demonstrated by Levänen *et al.* [20], individuals who are deaf have an enhanced ability to discriminate suprathreshold frequency changes in vibrotactile stimuli, which may support an enhanced ability to perceive vibrotactile musical frequencies.

Several studies have explored the human ability to discriminate timbres, such as different musical instruments, using vibrotactile stimuli alone. These studies have found that both deaf and hearing individuals are able to distinguish between dull and bright timbres [69]. Furthermore, deaf participants are able to distinguish between different voices singing the same phoneme on the same pitch [70]. It appears that this ability is owed to cortical integration of activity across the different channels of mechanoreceptors.

### 5.3. Assistive Technologies

In addition to the naturally occurring non-auditory aspects of music, music can also be manipulated through assistive technologies that are designed to enhance the experience of music. Various research groups are exploring the possibilities of using multi-modal technologies, including music visualizations and vibrotactile devices, to make music more accessible for the deaf population.

Though numerous visual representations of music have been created throughout the 20th century, including the iTunes visualizer and Walt Disney’s Fantasia, the effect of these models have not yet been well investigated with deaf participants. Changes in visual processing in the deaf population provide an opportunity for researchers to explore how visual moving patterns representing music might support an informative and enjoyable musical experience in deaf and hearing populations [71]. Music visualizations created for the deaf population might be best received in the periphery, while reserving central vision for unobstructed presentations of facial animation manifested by the performer.

Vibrotactile devices can enhance the experience of music for individuals who are deaf. Deaf individuals report that vibrotactile devices, such as haptic chairs, are a significant contribution to their musical enjoyment [72]. Using vibrotactile technology, music has been created in which vibrotactile aspects supersede the auditory aspects of music [73]. Research has shown that incorporating additional vibrotactile stimuli though sensory substitution technology may be an effective way to convey the emotional information when experiencing music [74,75].

In summary, a wealth of non-auditory information is available to provide a deaf audience with access to the structural and emotional aspects of music. The evidence that visual and vibrotactile stimuli may be processed in auditory centers of the brain supports the notion that non-auditory elements of music may provide an experience for deaf individuals that is qualitatively comparable to the experiences that a hearing person has while listening to music.

## 6. Music in the Deaf Community

Individuals who self-define as deaf span a wide range of hearing impairment from profound hearing loss (*i.e.*, dB HL (a logarithmic scale of sound intensity expressed in decibels and referenced to average normal hearing) of 91 or greater) to the maintenance of residual hearing (typically in the lower frequencies). The prevalence of signing and age of onset also varies. Because cross-modal plasticity is influenced by the extent of hearing impairment [22], signing and age of onset [38], the non-auditory enhancements they experience may also vary considerably [5]. The heterogeneity in the population may help to explain some of the mixed findings in the empirical results.

The majority of individuals in the deaf community report engagement in musical activities, such as singing/signing and moving or dancing to songs [76]. Novel forms of music that embrace vibrotactile and visual technologies are becoming increasingly popular, particularly among youth. For example, a method called “speaker listening” allows the user to feel the vibrations emitted from a speaker in order to experience the music in real time. Deaf discos provide a multi-sensory experience in order to make dance clubs more accessible for individuals who are deaf. A signed translation of the lyrics, also known as sign singing, has become widespread in the deaf community as a form of lyrical expression [76]. Professional sign language interpreters are hired in concert settings to provide a sign language rendition of the performances to deaf audience members.

Several examples of profoundly deaf and hearing-impaired musicians have confirmed that hearing is not a prerequisite for participating in musical activities. Beethoven is perhaps the most celebrated deaf musician. Beethoven continued to compose after the onset of his deafness by relying on the vibrations of his piano. A more recent example is Evelyn Glennie. Hearing impaired since 12 years of age, Gleenie is a virtuosic percussionist who relies on vibration heavily. She claims to feel “most musical sounds through the lower parts of (her) legs, feet, lower arms, wrists, chest, throat, and upper parts of (her) legs” [77].

It is also important to note that deaf individuals are increasingly being implanted with auditory prostheses known as cochlear implants (CI). The CI is a surgically implanted device that stimulates the auditory nerve via electrical impulses. Although CIs have opened up the world of music for deaf individuals, it has become increasingly clear that they do not perceive or process auditory aspects of music in the same manner as normal hearing individuals [78,79,80]. While individuals using CIs are able to perceive rhythm at a normal level, there is a clear deficit in pitch perception [81,82]. Nonetheless, the existence of accomplished musicians who use CI’s demonstrate that these deficits are not insurmountable.

## 7. Conclusions

There is abundant evidence for compensatory plasticity in individuals who are deaf. Specific enhancement of visual and vibrotactile skills in individuals who are deaf may provide differential processing of non-auditory aspects of musical performance. These enhancements may influence the manner in which music is perceived. Indeed, the definition of music need not focus on sound alone; music incorporates visual and vibrotactile information. Use of non-auditory modalities may be further enhanced through the use of assistive multi-modal technologies. The unique sensory abilities endowed by the deaf brain should be embraced when developing new music that is meant to be inclusive of the deaf community. These new works have the potential to advance the art of music for all.
